# Methods in PES-Learn: Direct-Fit Machine Learning of Born–Oppenheimer Potential Energy Surfaces

**DOI:** 10.3390/molecules31010100

**Published:** 2025-12-25

**Authors:** Ian T. Beck, Justin M. Turney, Henry F. Schaefer

**Affiliations:** Department of Chemistry, Center for Computational Quantum Chemistry, University of Georgia, Athens, GA 30602, USA

**Keywords:** machine learning, potential energy surface, kernel ridge regression, neural networks

## Abstract

The release of PES-Learn version 1.0 as an open-source software package for the automatic construction of machine learning models of semi-global molecular potential energy surfaces (PESs) is presented. Improvements to PES-Learn’s interoperability are stressed with new Python API that simplifies workflows for PES construction via interaction with QCSchema input and output infrastructure. In addition, a new machine learning method is introduced to PES-Learn: kernel ridge regression (KRR). The capabilities of KRR are emphasized with examination of select semi-global PESs. All machine learning methods available in PES-Learn are benchmarked with benzene and ethanol datasets from the rMD17 database to illustrate PES-Learn’s performance ability. Fitting performance and timings are assessed for both systems. Finally, the ability to predict gradients with neural network models is presented and benchmarked with ethanol and benzene. PES-Learn is an active project and welcomes community suggestions and contributions.

## 1. Introduction

The concept of a Born–Oppenheimer potential energy surface (PES) is fundamental to a multitude of chemical disciplines. An abundance of chemical information arises from the PES, including but not limited to reaction dynamics, kinetics, and geometric stationary-point information [1,2,3,4,5]. When attempting to model a PES, construction is often limited by the quantity of stationary-point information that is required. To alleviate the burden of informational requirements in PES construction, recent developments have turned toward fitting mathematical expressions to describe PESs in order to reduce the cost of construction [6]. In particular, machine learning (ML) methods have become popular for reducing the cost of modeling a PES [7,8,9,10,11,12,13,14,15,16]. A number of packages have been developed to facilitate the construction of ML models to accurately describe molecular PES and related chemical properties while reducing overall computational cost [17,18,19,20,21,22,23,24]. Several such packages have been highlighted in a recent special issue in *The Journal of Chemical Physics* for “Software for Atomistic Machine Learning” such as PANNA, MLIP-3, and DeePMD-kit, among others [25,26,27].

Machine learning methods that describe PESs are often broadly categorized into two groups. The first group attempts to describe the total energy of a system as the sum of atomistic energy contributions. These methods benefit in that they are able to describe an almost arbitrary sized system; however, these methods typically require a large amount of training data [17,28,29,30,31,32]. The second group of methods, which are the focus of this paper, aim to fit system-specific PESs directly. While atomistic methods are able to fit system specific PESs as well, the advantage of direct-fit methods is the fewer training points required and faster training time compared to atomistic methods [33]. The limitations, however, include the size of the system that is able to be studied (typically ≤ 10 atoms) and non-transferability to other systems.

Direct-fit ML has been repeatedly demonstrated as an accurate and reliable method for the construction of system-specific PESs. Popular ML methods that have utilized direct-fit include Gaussian process (GP) regression [34,35,36], various neural network (NN) methods [37,38,39,40,41,42,43], and kernel ridge regression (KRR) [7,44,45,46,47,48]. A recent study by Kushwaha and coworkers [37] benchmarked GP and NN methods available in PES-Learn. They examined the C_2_-He and NCCN-He PESs, restricting the training points of the sampled surface to less than 125 points, and they found root mean squared errors (RMSEs) of less than 1 cm^−1^ when compared to their exact dataset. In another study, Chmiela and coworkers [47] examined the use of KRR with gradient-domain ML (GDML) to fit PESs and predicted atomic forces of several intermediate-sized molecules with 1000 sampled configurations; they were able to produce a model accuracy of 0.3 kcal mol^−1^.

PES-Learn is a free open-source software package that automates the generation of datasets and ML model construction for the direct fitting of Born–Oppenheimer PESs [49]. With its inaugural paper, two classes of ML methods were available in PES-Learn: GP regression and feed-forward NNs. Here we introduce another popular ML method for modeling PESs, kernel ridge regression (KRR). This method has been shown to be reliable in a number of chemical applications including PES fitting [50,51,52], density functional theory (DFT) functional fitting [53,54], and prediction of chemical properties [55,56,57,58,59]. The interoperability and ease of use of PES-Learn are improved with the introduction of QCSchema support for dataset generation. Furthermore, the modularity of PES-Learn is reiterated via improved workflows and Python application programming interface (API) support. The version of PES-Learn utilized for this study was 1.0.1. The QCSchema project is developed by the Molecular Sciences Software Institute with the overarching goal of providing “API-like” inputs and outputs to a variety of quantum chemistry packages in a semi-standardized way [60].

In this paper, we introduce new capabilities of PES-Learn. New methods are presented, including the kernel ridge regression method for modeling PESs and the addition of gradient computations for trained neural network models via automatic differentiation. The ease of use of PES-Learn as a research tool is emphasized in the simplification of workflows and Python API support. Subsequently, the methods in PES-Learn are benchmarked with ethanol and benzene from the rMD7 database. Additionally, some examples from the previous PES-Learn paper are extended to the KRR method for comparison. Finally, the future directions of PES-Learn package are discussed with an invitation for community contributions. The PES-Learn software is open-source and freely available on GitHub (https://github.com/CCQC/PES-Learn (accessed on 1 June 2023)).

## 2. Discussion

Generating an ML model of a PES for a particular molecular system is largely automated with the PES-Learn package, from data generation to model construction. The present section outlines the improvements that have been made to PES-Learn in order to make this process easier and more interoperable, in addition to highlighting the new methods available. In addition to new workflows and methods, the PES-Learn documentation has been reinvented to include additional examples, more robust descriptions, and suggested workflows.

### 2.1. Dataset Generation and Sampling

The PES-Learn workflow can generally be divided into four parts: (1) constructing a dataset of PES state points; (2) parsing the data and compiling a dataset; (3) building a machine learning model; and (4) working with the model. This section covers updates to the first two steps, while the next section covers the last two.

PES-Learn supports the use of external datasets (provided that they are in a readable format), but it is often desirable for users to generate their own custom datasets with tools provided by PES-Learn. The previous workflow for generating datasets was based on creating a template file that modeled the input for an electronic structure theory (EST) program of choice in addition to a PES-Learn input file. The template file contained all the necessary keywords and inputs for the preferred EST program, while the PES-Learn input file contained the PES-Learn inputs as well as a standard Z-matrix of internal coordinates along with ranges by which the internal coordinates would be defined for the dataset. PES-Learn then generated input files for the EST program with geometries over the range of defined coordinates. This legacy workflow is still available to use.

The new workflow condenses inputs to one file that is read by PES-Learn. This new workflow is made possible with the use of QCSchema input and output objects. A user sets the ‘schema_generate’ keyword to ‘True’ to initiate the generation of QCSchema inputs. Additionally, the user specifies the EST program of choice (as long as it is supported by QCSchema), the method of computation, the basis set, and any other keywords to pass to the EST program. With these keyword specifications, PES-Learn generates Python scripts that contain all the necessary information to generate a QCSchema input object to run with QCEngine in a similar manner so that it generates input files from templates. This simplified workflow with an improved Python API stresses the importance of interoperability. The QCSchema interface eases the ability to interact with a variety of EST software packages.

Previous studies have examined a variety of methods to reduce the size of the grid of data points required to construct a PES [61,62]. It is essential to employ a strategy to reduce the number of grid points before generating data, and several options exist within PES-Learn to modify the sampling of grid points. These methods aim to maximize the area spanned by the PES and minimize the computational cost of construction. The option to remove symmetry redundant geometries that are generated from the range of internal coordinates is available in PES-Learn to guarantee that only unique symmetrical geometries are used. PES-Learn does this by incorporating permutational symmetry with fundamental invariants (FIs) of permutation-invariant polynomials (PIPs). When identical atoms in a molecule are permuted such that geometry is preserved, for example when the two hydrogens in a water molecule are swapped, the model output should remain unchanged regardless of which geometry is used as the input. To incorporate this permutational symmetry, a minimal set of PIPs of interatomic distances are used [63,64]. The PIPs are invariant under permutational symmetry, so they are used as geometric inputs for an ML model instead of other representations of geometric information such as simple internal coordinates. The minimal sets of these PIPs are called FIs [65,66]. PES-Learn contains a library of these FIs to aid in data transformation. The use of PIPs and FIs has been shown to improve both the accuracy and speed of generating PESs when compared to other methods, particularly with popular programs such as PESPIP and PIPFit [67,68,69,70,71]. A more detailed discussion of PIPs and FIs and their application to chemical machine learning has been carried out in the early PES-Learn paper and elsewhere [49,72]. Additionally, PES-Learn allows grid size specification with the ‘grid_reduction’ keyword. By specifying the size of the grid, PES-Learn will reduce the number of points in the grid to the specified integer by maximizing the Euclidean distance between points. This is performed after symmetry redundancies are removed, if requested.

**Figure 1:**
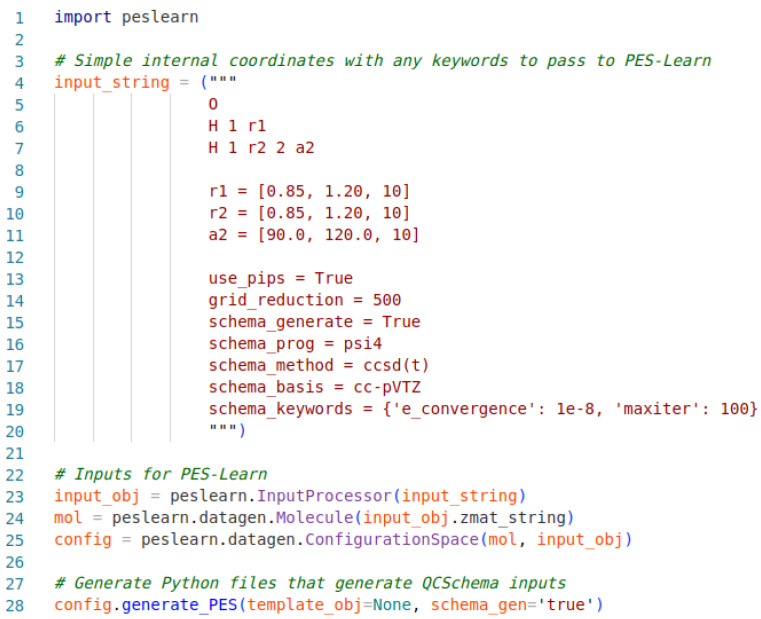
Sample Python input file structure for generating QCSchema inputs/outputs with PES-Learn.

An example PES-Learn Python input file for the generation of QCSchema inputs is given in Figure 1. The input file begins by importing the PES-Learn Python package, peslearn. Following this is an input_string first containing a Z-matrix of simple internal coordinates. In this example, the sample molecule is water, and the internal coordinates for this system are defined as the bond lengths between O and each H, r1 and r2, and the bond angle between the three, a1. The internal coordinate ranges are defined by the lists immediately following the Z-matrix with the syntax [first value, final value, number of points]. The number of points will inform PES-Learn how many geometries to generate. Since there are three simple internal coordinates in the example system, each defined with ten points, this will generate 10^3^ geometries. The next section in the input string involves keywords; the first two keywords are for reducing the grid of geometries. The ‘use_pips’ keyword enables the use of PIPs to remove symmetry-redundant geometries, and ‘grid_reduction’ reduces the size of the geometry grid after removal of redundancies.

The remaining keywords in the input string describe how to build the QCSchema inputs. Generation of QCSchema inputs is toggled with the ‘schema_generate’ keyword, and ‘schema_prog’ tells QCEngine what EST program to run—Psi4 in the example case. The keywords ‘schema_method’, ‘schema_basis’, and ‘schema_keywords’ define what QCEngine passes on to the EST program stated previously. These keywords should be set according to the EST program being utilized; for example, whatever is passed into ‘schema_keywords’ in the given example must be interpretable by Psi4 as a valid keyword. In the given example, the potential energy surface for water will be generated from CCSD(T)/cc-pVTZ energies. This is a purely illustrative example and, as always, the machine learning model is only as good as the dataset used to generate it. Lines 23–25 allow PES-Learn to interpret the input and utilize it in line 28, which generates the Python scripts to run QCEngine and generate QCSchema inputs and outputs.

After geometric data are generated and energies are computed, the parsing of these energies is required for the next step, which will be ML modeling. PES-Learn automatically handles the parsing of these energies in several ways. Parsing of energies with the cclib package or Python regular expressions has been implemented previously and will not be discussed here. New to PES-Learn is the automatic parsing of energies from QCSchema output objects. After computations are finished running via QCEngine, the QCSchema output is written to an output file. Regardless of the EST program that QCEngine utilizes, the output schema handles all of the parsing automatically into a standardized JSON structure. By telling PES-Learn to parse from QCSchema, it will find the standard output automatically and generate a dataset containing all the energies and geometric information, without further input from the user.

Building an ML model after data collection requires the partitioning of data into training and test sets. The training dataset is used to build and optimize the model, while the test set is not seen by the model until after training and is used purely for performance validation. There are no strict guidelines on how to partition the data; ideally, the training set should be holistically representative of the dataset so the trained model is able to make accurate predictions for data points in-between training points. The training set should be sampled in a way that the data points within encompass a diverse range of geometries in order to make accurate predictions on new data. PES-Learn provides the means to partition datasets automatically in a number of ways. From the previous version of PES-Learn, smart random and structure-based sampling are both still available. Random sampling is a common method used to partition data for many ML applications; however, for generating PESs, this can often leave “holes” in the potential surface where the model has not been trained. An alternative to random sampling is smart random sampling. This method of sampling chooses a random seed to partition the training set so it most closely resembles the entire dataset, based on the chi-squared test [73]. The implementation of chi-squared test comparisons ensures that any outlier data point will not be included in the training set, as long as it is not represented by the overall dataset. Structure-based sampling maximizes Euclidean distances between geometries of training points and creates a training set of evenly spaced points [44].

A new sampling method incorporated into PES-Learn is Sobol’ sampling [74]. This method is a quasi-random sampling of training points in accordance with their relative energies. The PES-Learn implementation of Sobol’ sampling is based on the previous implementation of Manzhos and Carrington [75]. With this sampling method, an expression is derived from the relative energy, as shown in Equation (1).

(1) $$\frac{E_{max,rel}-E+\delta }{E_{max,rel}+\delta }$$

In this equation, $E_{max,rel}$ is the maximum energy relative to the minimum energy of the dataset ($E_{max}-E_{min}$), *E* is the energy of the data point being considered for the training set, and δ is a shifting factor defaulted to 500 cm^−1^, which biases the dataset toward low- to mid-range energies. The data point corresponding to *E* is appended to the training set if this metric in Equation (1) is greater than a random number between 0 and 1. The training set that is generated from this sampling is not representative of the whole dataset but is instead biased toward low- to mid-range energies. This may be useful, for example, in modeling minima for a vibrational application such as vibrational configuration interaction computations.

### 2.2. Methods and Models

Gaussian process regression and feed-forward neural networks have been available in PES-Learn since its initial release. We now introduce the ability to construct ML models for direct-fit PESs with kernel ridge regression (KRR). KRR is an ML method that implicitly maps data to a feature space in order to learn a relationship in the data. The implicit mapping is performed without having to know the transformation to the feature space, which allows for a great reduction in the computational cost. By implicit transformation into some feature space, a relationship may be learned that is not as apparent in the space of the input. The details and formalisms of KRR and its relations to PESs have been described elsewhere [54,76,77]. KRR models are created via interface in scikit-learn, a Python library containing many popular ML algorithms [78]. The scikit-learn package allows for several options of kernel functions with KRR; in PES-Learn, the choice of this kernel is a hyperparameter by default.

Hyperparameters are some criteria or variables in an ML model that are set before model training. An example of this could be kernel choice or data-scaling method. Choice of hyperparameters is crucial when building an ML model, and changing even one could drastically affect model’s performance. Ideally, to find the best hyperparameters, one would search the entire grid of possibilities and find the ones that perform the best. Unfortunately, the size of the hyperparameter space is often too large to conceivably test every possibility. To aid in hyperparameter optimization, PES-Learn utilizes the popular HyperOpt package to find correlations between hyperparameters and model performance. With sufficient iterations, depending on the size of the hyperparameter space, HyperOpt is fairly reliable in minimizing model RMSE. PES-Learn gives an option for specification of the hyperparameter space if a user ascertains that some hyperparameters work better than others.

Hyperparameter optimization for the three different ML methods in PES-Learn currently requires a scan over drastically different hyperparameter spaces. Each method has different hyperparameters and, as such, spans different spaces. There are approximately two to three times as many hyperparameter options for NNs as there are for GP and approximately 100 times as many possibilities for KRR than there are for NNs. The consequence of this is that KRR requires many more hyperparameter optimization iterations than the other two methods. However, we show that in the PES-Learn implementations, the computational cost of KRR is much smaller than GP or NNs. This is illustrated in the examples in the next sections; some KRR timings are given and compared against timings for GP and NNs to give a sense of optimization scaling between the methods as they have been implemented. While GP regression and KRR are strikingly similar, the two methods are unique in construction. The differences in GP regression and KRR, as they relate to model assumptions and convergence rates, have been previously examined [79].

After hyperparameter optimization and model training, PES-Learn saves the model with the best performance to be used for further application. PES-Learn writes a Python function from the saved model to then predict energies of a given geometry. If PES-Learn has performed any data transformation during model training, it saves that in the Python function, so that any transformation will be handled again automatically, without input from the user.

Another new method introduced in PES-Learn is the ability to predict gradients with a constructed neural network model. This is performed via an interface in PyTorch, the same framework that PES-Learn utilizes for building NN models. PyTorch provides foundations upon which to perform automatic differentiation (auto-diff). Auto-diff in PyTorch is achieved by constructing a computational graph as mathematical operations are performed through a model, which can then be followed backward by using derivative chain rules to compute a gradient automatically [80]. When an NN model is constructed in PES-Learn, the output Python function can now be utilized to compute the energy of a given geometry and also the force as the negative gradient of the energy with respect to input coordinates. Predicting forces with an ML model necessitates the consideration of energy conservation [55]. When predicting a force directly, e.g., as the output of an ML model, special considerations must be made to ensure the predicted forces are energy-conserving and, by extension, obey the Hellmann–Feynman theorem [81]. The advantage of predicting forces by differentiation is that they are energy-conserving by construction.

## 3. Results

### 3.1. Examples Revisited

In the previous PES-Learn paper, the fitting performance of GP and NN models created with PES-Learn was examined and compared to the performance of preexisting models in the literature. The ability of PES-Learn to create models in an automated fashion that were able to perform as well as or better than previous models was demonstrated. Here, we extend this examination to PES-Learn’s new KRR method and compare KRR’s fitting performance to select previous models and to PES-Learn’s GP and NN models.

All models created with PES-Learn were generated automatically with PES-Learn’s built-in protocol; no hyperparameters were set explicitly by the user. Additionally, internal coordinate geometries were transformed with fundamental invariants to preserve permutational symmetry. Three potential surfaces were compared, with varying sizes of ab initio training sets and sampling methods. The surfaces, H_3_O^+^, OCHCO^+^, and H_2_CO, are from a study by Bowman and coworkers that compared GP regression to a linear least-squares PIP (PIP-LS) approach [82]. The H_3_O^+^ surface, first published by Yu and coworkers [83], contains 32,141 energy geometry pairs over a span of approximately 21,000 cm^−1^. This surface spans two $C_{3v}$ minima separated by a $D_{3h}$ saddle point. The OCHCO^+^ surface is described as a hydrogen transfer between two CO groups via a $D_{\infty h}$ saddle point and spans approximately 22,000 cm^−1^ with 7800 data points, first published by Fortenberry and coworkers [84]. The final surface is for the isomerization of H_2_CO to *cis*- and *trans*-HCOH via two separate saddle points. This surface covers approximately 50,000 cm^−1^ and 34,750 data points.

These paradigm surfaces span multiple minima and saddle points, among other surface topography. When building a dataset with PES-Learn, it is important to cover a range of internal coordinates such that it will span the important and desired topography on the PES. A prime example of this is present in the surfaces that have been examined here. The OCHCO^+^ surface portrays two minima separated by a saddle point. The minima on this surface are connected as the hydrogen transfers between the two CO groups. Generation of internal coordinates for this surface with PES-Learn would need to include a broad range of C-H distances in combination with variation of other internal coordinates.

Table 1 compares the RMSE performance of models from a previous study by Bowman and coworkers [82] to GP, NN, and KRR models generated with PES-Learn. The GP and NN models made with PES-Learn are the ones presented in the previous PES-Learn paper [49]. The GP and NN models were allowed to cycle through 20 hyperparameter optimization iterations, while KRR was allowed 200 to account for its larger hyperparameter space. Columns labeled as the reference sample (RS) used the exact same training and test set as in the reference models [82]. This allowed for a direct comparison of other models to PES-Learn models. The columns labeled structure-based (SB) correspond to PES-Learn choosing a training set achieved with the structure-based sampling method described above, keeping the number of training and test points consistent with the previous study’s models. Similarly, smart random (SR) columns correspond to smart random sampling of training set as described above, again with the same training and test set partitioning sizes.

The models constructed with KRR performed the best for the H_3_O^+^ surface. All but two of the automatically generated models outperformed the RMSE of the models from the previous paper. The SB sampling routine produced models with substantially better fitting performance than the models generated with SR sampling or the sampling of the reference. The model trained on 1000 points with SB sampling found an RMSE of 24.4 cm^−1^, a smaller RMSE than both the PIP-LS and GP models from the previous paper trained on 2000 points with RMSEs of 28.1 and 36.3 cm^−1^, respectively.

According to KRR for the other two surfaces, OCHCO^+^ and H_2_CO, did not perform as extraordinarily as the H_3_O^+^ surface.All of the models produced with SB sampling did perform better than both previously published models, apart from H_2_CO with 5104 training points. Additionally, all of the KRR models for OCHCO^+^ and H_2_CO yielded better RMSE performance than the PIP-LS models with their respective number of training points.

**Figure 2:**
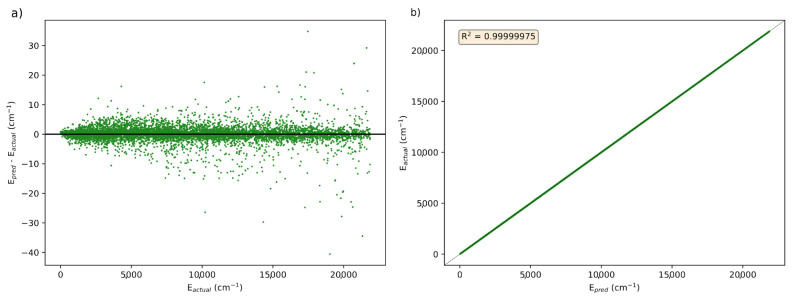
(**a**) Comparison of the prediction errors ($E_{pred}-E_{actual}$) versus known energies ($E_{actual}$) in cm^−1^ for the KRR model of OCHCO^+^ trained on 1560 points with a structure-based sampling system. (**b**) Known energies versus predicted energies for the KRR model of OCHCO^+^trained on 1560 points with a structure-based sampling system.

After building a model, PES-Learn generates a Python function to predict energies based on the trained model. Using this function, predictions can be made on geometries from the full dataset and compared against their known energies. Figure 2a plots the prediction error, represented as $E_{pred}-E_{actual}$ (in cm^−1^), versus actual energies, $E_{actual}$ (in cm^−1^), for the best performing KRR model from Table 1. This model was from the OCHCO^+^ surface with 1560 training points using a structure-based sampling regime. The distribution of errors centered around 0 cm^−1^ demonstrates PES-Learn’s ability to create accurate ML models with the new KRR method. Figure 2b plots the actual versus predicted energy from the PES-Learn-generated Python function to examine the variance in the predicted energies from actual. There is very little variance from the actual energies, which can be seen in how closely the data points are to the black dashed line that is 1:1. The R^2^ value, or coefficient of determination, numerically shows the variance between actual and predicted values. An R^2^ value of 1 means that the data match exactly; the R^2^ for the energies predicted from this model is 0.99999975, further demonstrating that the predicted energies are very close to the actual energies.

KRR models produced with PES-Learn did not outperform PES-Learn’s GP or NN methods. However, the important thing to note is the extreme cost reduction that came with using KRR, compared to GP or NN methods. All of the models produced with PES-Learn were run in parallel across four cores on an old Intel Xeon E3-1270 v5 @ 3.60 GHz CPU, a CPU that was released just over ten years ago. Most of the GP and NN models were built in under 8 h, with most smaller training sets building in less than 30 min. All KRR models were built in around 20 min or less, with smaller training sets taking less than 5 min. The exceptions are for particularly large datasets with more than 5000 training points, which took around 2 h.

It should be noted that the results for models built with KRR in Table 1 can be greatly improved with more robust hyperparameter optimizations. To get a sense of how well KRR can do, additional models were created without specifying the partitioning of training and test sets beyond requesting SB sampling. One model for each surface was generated and allowed to run for 1000 hyperparameter optimization iterations, which is still considerably small compared to the extent of the hyperparameter space of KRR. The models for H_3_O^+^, OCHCO^+^, and H_2_CO yielded RMSEs of 9.5, 0.4, and 112.7 cm^−1^, respectively. These models were still built in an automated fashion, meaning no hyperparameters were explicitly set. Comparing the error in these models to the ones generated in the previous study by Bowman and coworkers [82], the H_3_O^+^ model is only outperformed by the GP model with 10,000 training points and the PIP-LS model trained on the entire dataset. The models for OCHCO^+^ and H_2_CO produce lower errors than any of the original PIP-LS or GP models. Further optimization while specifying hyperparameters that work well would greatly improve the KRR results in Table 1.

### 3.2. Benzene

The next two sections present benchmark data to explicate the accuracy of the current methods implemented in PES-Learn. The rMD17 database [47,85] contains datasets that are designed and often employed for assessing methods that describe potential energy surfaces [86,87]. The datasets within the rMD17 database are described by 100,000 geometries from direct dynamics simulations and corresponding energies computed using density functional theory with the PBE functional [88] and the def2-SVP basis set [89]. In this section, numerical errors, as RMSEs, and timings are presented for each of the three ML methods available in PES-Learn when run on the benzene and ethanol datasets from the rMD17 database. Additionally, each of the ML methods has been examined with each of the three training/test set sampling methods available. Unless specified otherwise, all of the models constructed in this section were built in a completely automated fashion, and no hyperparameter tuning was carried out by the user.

**Figure 3:**
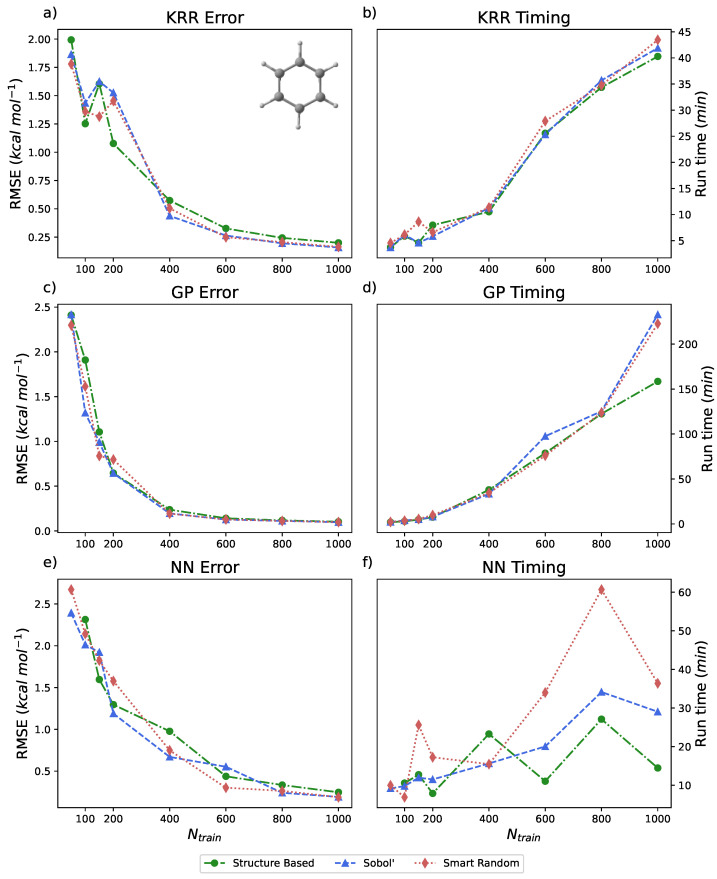
(**a**,**c**,**e**) Root-mean-squared-errors (RMSEs) (in kcal mol^−1^) for KRR, GP, and NN methods, respectively, available in PES-Learn benchmarked with benzene from the rMD17 database. (**b**,**d**,**f**) Timings (in minutes) for PES-Learn execution when constructing KRR, GP, and NN models, respectively, on benzene from the rMD17 database.

The energy range for benzene in the rMD17 dataset is approximately 20 kcal mol^−1^. All of the models built for this benchmark did not utilize PIPs; the number of inputs is reflective of the simple internal coordinates of benzene, of which there are 65. As illustrated in Figure 3a,c,e, each ML method in PES-Learn achieves relatively low RMSE. Data is also presented in Table 2. Note that using only 1% of the dataset across all models and sampling types, the RMSE is less than 0.25 kcal mol^−1^. The study that originally published the rMD17 database examined the mean absolute errors (MAEs) of learning curves trained on kernels constructed from atomic environments [85]. In their examination of benzene, the MAEs for their learning curves present in the 0.01−0.1 kcal mol^−1^ range for the same number of training points ($N_{train}$) presented here. The difference in strategies for constructing models (atomistic versus direct-fit) and the difference in error metric (MAE versus RMSE) should be noted. In particular, RMSE is more sensitive to outlier data points [90], which are abundantly present in this dataset in the form of numerical noise [86,91]. The timings for these methods are shown on the right-hand side of Figure 3b,d,f. These timings were carried out by running PES-Learn in parallel across four cores on an Intel Xeon E3-1270 v5 @ 3.60GHz CPU, and we measured the entire PES-Learn run time from partitioning training sets to saving the ML model. The reasoning behind the sporadicity of the NN timings is the size of the NN on which models are trained. Each model chooses different parameters for depth and number of hidden layers based on hyperparameter search, and the architecture of the final NN will greatly affect the timing of cross-validation.

### 3.3. Ethanol

**Figure 4:**
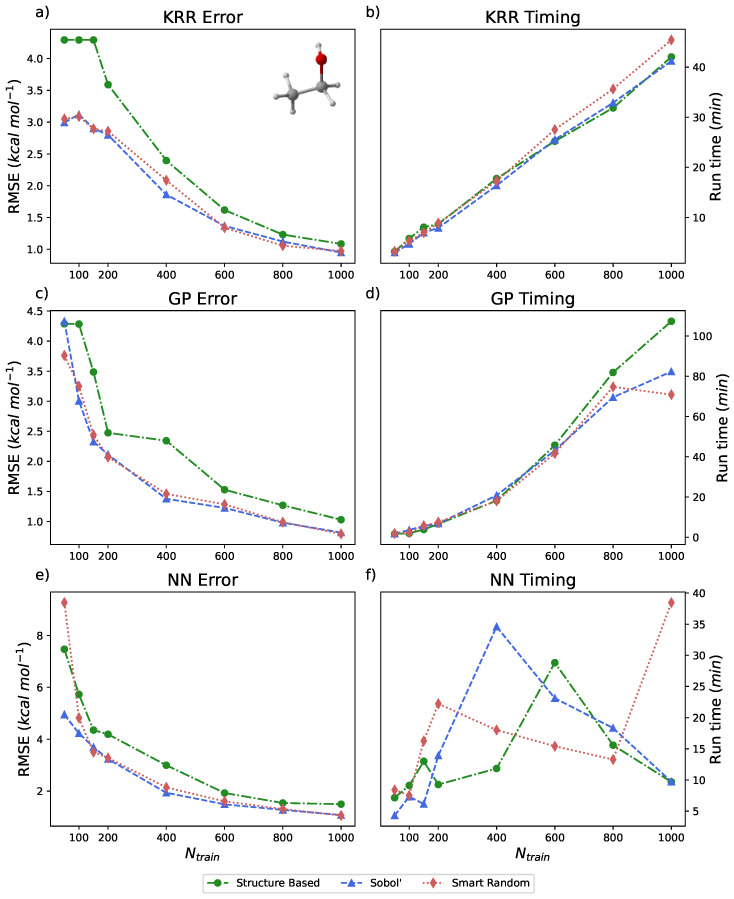
(**a**,**c**,**e**) Root mean squared errors (RMSEs) (in kcal mol^−1^) for KRR, GP, and NN methods, respectively, available in PES-Learn benchmarked with ethanol from the rMD17 database. (**b**,**d**,**f**) Timings (in minutes) for PES-Learn execution when constructing KRR, GP, and NN models, respectively, on ethanol from the rMD17 database.

The ethanol dataset from the rMD17 database is represented by 100,000 energy–geometry pairs that span approximately 25 kcal mol^−1^. Models trained on ethanol with PES-Learn did not incorporate PIPs; the number of simple internal coordinates used as inputs for model training with ethanol was 35. The results of running PES-Learn with this dataset, as RMSEs, are presented in Figure 4 and Table 3. The RMSEs from these PES-Learn constructed models fare well. Similar to the benzene dataset, almost all of the constructed models yield relatively small errors with only 1% of the total dataset. The RMSEs presented here range from 1 to 2 kcal mol^−1^ with 1000 training points. Another study [87] examined the performance of eight different models trained on the ethanol dataset. Their examination revealed that most of the models they tested also yielded RMSE values in the 1–2.5 kcal mol^−1^ range for the same number of training points. This reinforces the fact that PES-Learn-constructed models are on par with other models in the literature. Timings for PES-Learn run on the ethanol dataset are presented in Figure 4b,d,f, which were executed in the same manner as benzene. Timings reported in Figure 3 and Figure 4 constitute the entire PES-Learn runtime. The majority of the runtime is hyperparameter optimization followed by model fitting/validation. Approximately 99% of the GP and KRR runtime is taken up by hyperparameter optimization, whereas the NN hyperparameter optimization varies between 75 and 90% depending on model architecture.

### 3.4. Gradients

**Figure 5:**
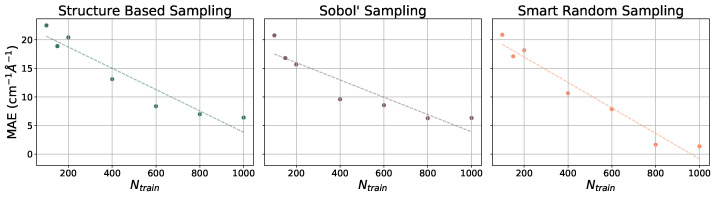
Learning curves for models in PES-Learn when predicting gradients on ethanol from the rMD17 database. Mean absolute errors (MAEs) are in cm^−1^ Å^−1^. Dotted lines are lines of best fit.

**Figure 6:**
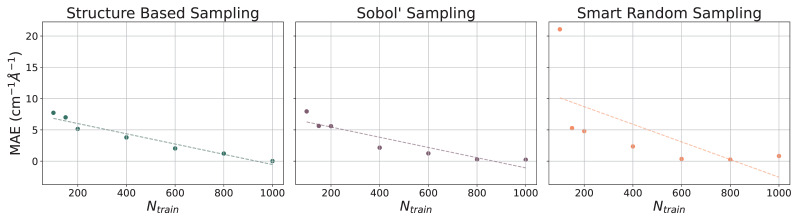
Learning curves for models in PES-Learn when predicting gradients on benzene from the rMD17 database. Mean absolute errors (MAEs) are in cm^−1^ Å^−1^. Dotted lines are lines of best fit.

The ethanol and benzene datasets were also utilized for gradient benchmarking. The machine learning models used to predict gradients are all NN models with various training set sizes ($N_{train}=\left\{100,150,200,400,600,800,1000\right\}$). These models incorporate hand-selected hyperparameters, unlike the models that have been presented thus far. The learning curves for the prediction of gradients for the ethanol dataset are illustrated with MAEs (in cm^−1^ Å^−1^) in Figure 5 and for the benzene dataset in Figure 6. As the number of training points increases when constructing each model, the MAE decreases at a relatively steady rate. For both paradigm molecules, using all three sampling methods, the MAE is converged at or close to 5 cm^−1^ Å^−1^ by $N_{train}$ = 1000, which is only 1% of the dataset. Trends in the learning curves suggest that further increasing the number of training points would continue to decrease the MAE in predicted gradients. It has previously been observed that the use of more than 1000 training points from the rMD17 database would include numerical noise as a result of redundancies in geometric information in the datasets [86,91]. A more robust dataset would provide a means of helping to decrease the noise and further decrease the error.

### 3.5. Future Directions

The development of PES-Learn is active, with ideas being discussed to improve this software and make it a superb tool for the automatic generation of ML models for PESs. It has been suggested that the inclusion of gradients or Hessians in training data for modeling molecular PESs would drastically improve the performance of the model [85,92,93,94,95]. The use of gradients is available in other ML PES software such as PESPIP [67], and the inclusion of gradients/Hessians in PES-Learn when building a model could expand this ongoing research. While PES-Learn is currently being used to describe adiabatic PESs, the inclusion of nonadiabatic dynamics is often important for describing systems where the Born–Oppenheimer approximation breaks, such as a surface crossing. Efforts in ML have been made to distinguish nonadiabatic surface data from adiabatic surface data, such as diabatization by a deep neural network (DDNN), as proposed by Truhlar [96]. The inclusion of methods to describe nonadiabatic effects would improve the ability to describe systems where this is important. Another improvement would be the inclusion of more ML methods. An eventual goal is to add more types of ML methods that are popular and have been shown to work well in building molecular PES models. Different types of NNs have proved popular in the recent literature, such as multifidelity methods [97], which mix small high-accuracy datasets with large low-accuracy datasets to build models with high accuracy at lower computational cost. The inclusion of a method to sample the conformational space of a system would allow users to have no a priori knowledge of system minima. This would allow for a reduction in the number of points needed to describe local minima on a PES. Further improvement in usability would involve workflow changes that make it easier to generate data and build models, but the old workflows would remain, allowing users to individually decide how they use PES-Learn. The limitations on PES-Learn’s ability to scale to global PESs are largely imposed by computational resources. To improve the scalability of PES-Learn when handling particularly large datasets, it would be desirable to add support for graphics-processing units (GPUs) and threading capabilities. Improving computational scaling via these methods would allow for increased scaling beyond semi-global PESs to the study of global surfaces.

## 4. Conclusions

The software package PES-Learn is constructed and incorporated in the full 1.0 release. Illustrated in this paper are the capabilities of the kernel ridge regression (KRR) method and many other new features added to PES-Learn. The ability to generate QCSchema inputs and outputs strengthens PES-Learn as an interoperable software package by enabling simple workflows to interact with a variety of electronic structure theory packages. An emphasis on PES-Learn’s automation for generating machine learning models of semi-global molecular potential energy surfaces is shown in the models that were constructed in a completely automated fashion. The fitting performance of PES-Learn’s new KRR method was examined and found to be comparable to other machine learning methods in PES-Learn with previous applications; it was compared using benzene and ethanol datasets from the rMD17 database. We found that the new KRR method was able to produce machine learning models able to outperform models in previous studies, and it significantly reduced the time needed for model construction. A benchmark of all methods available in PES-Learn is presented. The fitting performance and timings for PES-Learn model construction were measured for benzene and ethanol from the rMD17 database. The fitting performance of PES-Learn with these datasets is compared herein to other models in the literature, and PES-Learn is shown to be equal to these models. The ability to predict gradients with neural network machine learning models in PES-Learn is also presented. Mean absolute error learning curves show that PES-Learn is able to compute gradient data with an accuracy similar to other software packages, with a small amount of training data. PES-Learn is in active development and has many features envisioned for the future; we continue to welcome community contributions and suggestions. PES-Learn software is open-source and freely available on GitHub (https://github.com/CCQC/PES-Learn (accessed on 1 June 2023)).

## Figures and Tables

**Table 1:** Fitting errors measured as RMSE (in cm^−1^) for H_3_O^+^, OCHCO^+^, and H_2_CO PESs using five methods of fitting: linear least-squares fitting of permutationally invariant polynomials (PIP-LS) and the Gaussian processing (GP) models reported by Bowman et al. ^*a*^ PES-Learn automatically generated kernel ridge regression (PES-Learn KRR) model, and PES-Learn automatically generated Gaussian process (PES-Learn GP) and neural network (PES-Learn NN) models reported by Abbot et al. ^*c*^. Errors are given for different test and training sampling schemes; reference sample (RS) is the exact partitioning reported by Bowman et al. ^*a*^ Structure-based (SB) and smart random (SR) are schemes included in PES-Learn.

		PIP-LS ^*a*^	GP ^*a*^	PES-Learn KRR ^*b*^			PES-Learn GP ^*c*^			PES-Learn NN ^*c*^		
	$N_{\mathrm{train}}$	RS	RS	RS	SB	SR	RS	SB	SR	RS	SB	SR
H_3_O^+^	500	116.99	238.74	85.01	51.22	91.68	19.97	15.24	22.59	22.53	15.27	34.76
	1000	39.20	125.76	40.40	24.38	42.14	10.71	6.09	10.22	13.99	11.77	27.78
	2000	28.13	36.33	23.59	17.38	22.52	5.88	2.94	6.36	11.35	6.99	15.31
	5000	11.81	11.17									
	10,000	9.99	5.62									
	32,141	7.09										
OCHCO^+^	520	259.18	32.09	53.88	25.82	58.59	23.77	8.70	19.62	38.84	14.33	46.79
	780	293.53	26.62	37.34	21.86	49.79	23.03	4.06	17.87	29.60	5.92	34.96
	1560	128.65	15.78	27.25	2.82	19.83	10.58	1.03	8.03	11.99	2.33	10.30
	2600	97.40	15.55									
H_2_CO	5104	432	238	228.28	260.50	358.82	191.15	402.84	375.27	118.53	124.09	189.14
	8703	332	229	234.38	201.00	290.50				121.07	91.36	146.29

^*a*^ From Ref. [82]; ^*b*^ From this research; ^*c*^ From Ref. [49].

**Table 2:** Benchmark data for all models (kernel ridge regression (KRR), Gaussian process (GP), feed-forward neural networks (NN)) and sampling methods (structure-based (SB), Sobol’, and smart random (SR)) available in PES-Learn, run on benzene from the rMD17 database.

		KRR			GP			NN		
	$N_{\mathrm{train}}$	SB	Sobol’	SR	SB	Sobol’	SR	SB	Sobol’	SR
RMSE (kcal mol^−1^)	50	1.99	1.86	1.78	2.41	2.42	2.30	41.18	2.39	2.67
	100	1.25	1.43	1.36	1.91	1.32	1.61	2.31	2.01	2.14
	150	1.61	1.63	1.31	1.11	0.99	0.84	1.60	1.92	1.82
	200	1.08	1.53	1.45	0.65	0.64	0.80	1.30	1.19	1.58
	400	0.57	0.44	0.50	0.24	0.20	0.19	0.98	0.67	0.75
	600	0.33	0.26	0.25	0.14	0.13	0.13	0.44	0.55	0.30
	800	0.24	0.20	0.21	0.12	0.11	0.11	0.33	0.24	0.26
	1000	0.20	0.16	0.16	0.10	0.10	0.10	0.25	0.19	0.19
Runtime (min)	50	3.72	3.70	4.56	1.86	2.19	2.18	5.60	9.17	9.96
	100	5.87	6.14	6.16	2.88	3.93	3.52	10.55	9.76	6.88
	150	4.61	4.59	8.62	4.77	4.93	5.42	12.72	11.95	25.60
	200	7.98	5.83	6.60	7.46	7.95	9.79	7.87	11.49	17.23
	400	10.54	11.27	11.35	37.94	33.38	34.50	23.28	15.63	15.45
	600	25.57	25.31	27.90	78.68	97.50	75.94	11.03	20.04	34.00
	800	34.36	35.71	34.75	122.51	125.31	123.77	27.10	34.13	60.67
	1000	40.29	41.88	43.46	158.49	232.81	222.74	14.46	29.03	36.38

**Table 3:** Benchmark data for all models (kernel ridge regression (KRR), Gaussian process (GP), and feed-forward neural networks (NN)) and sampling methods (structure-based (SB), Sobol’, and smart random (SR)) available in PES-Learn, run on ethanol from the rMD17 database.

		KRR			GP			NN		
	$N_{\mathrm{train}}$	SB	Sobol’	SR	SB	Sobol’	SR	SB	Sobol’	SR
RMSE (kcal mol^−1^)	50	4.29	2.99	3.05	4.29	4.33	3.76	7.47	4.95	9.27
	100	4.29	3.12	3.09	4.28	3.01	3.25	5.73	4.23	4.82
	150	4.29	2.90	2.90	3.49	2.33	2.44	4.36	3.70	3.51
	200	3.59	2.80	2.86	2.47	2.11	2.07	4.19	3.24	3.28
	400	2.40	1.86	2.08	2.34	1.38	1.46	3.00	1.94	2.14
	600	1.62	1.37	1.34	1.53	1.23	1.28	1.93	1.49	1.61
	800	1.23	1.12	1.06	1.27	0.98	0.99	1.54	1.27	1.31
	1000	1.08	0.95	0.97	1.03	0.82	0.79	1.50	1.08	1.06
Runtime (min)	50	3.19	2.99	3.22	1.66	1.74	1.87	7.14	4.30	8.39
	100	5.78	4.72	5.32	1.93	3.59	2.24	9.11	7.30	7.51
	150	8.05	6.96	6.98	3.85	5.57	5.72	13.00	6.16	16.27
	200	8.67	7.91	8.87	6.60	6.77	7.51	9.27	13.92	22.21
	400	17.75	16.37	17.27	18.17	20.80	18.03	11.84	34.59	18.03
	600	25.21	25.52	27.54	45.76	43.13	41.66	28.81	23.09	15.42
	800	31.85	32.88	35.62	81.92	69.55	74.74	15.59	18.34	13.28
	1000	42.03	41.21	45.42	107.33	82.34	70.86	9.69	9.72	38.47

## Data Availability

The PES-Learn code used in the study is openly available at https://github.com/CCQC/PES-Learn (accessed on 1 June 2023). The version of the code employed for this study is version 1.0.1.

## Abbreviations

The following abbreviations are used in this manuscript:

PES	potential energy surface
ML	machine learning
GP	Gaussian process
NN	neural network
KRR	kernel ridge regression
RMSE	root mean squared error
DFT	density funcitonal theory
API	application program interface
EST	electronic structure theory
PIP	permutationally invariant polynomial
FI	fundamental invariant
RS	reference sample
SB	structure-based
SR	smart random
MAE	mean absolute error
